# Self-entrustment: how trainees’ self-regulated learning supports participation in the workplace

**DOI:** 10.1007/s10459-016-9723-4

**Published:** 2016-10-26

**Authors:** Margaretha H. Sagasser, Anneke W. M. Kramer, Cornelia R. M. G. Fluit, Chris van Weel, Cees P. M. van der Vleuten

**Affiliations:** 1 Department of Primary and Community Care, Radboud University Medical Centre Nijmegen, Nijmegen, The Netherlands; 2 Department of Public Health and Primary Care, Leiden University Medical Centre, Leiden, The Netherlands; 3 Centre on Research in Learning and Education, Radboud University Medical Centre Nijmegen, Nijmegen, The Netherlands; 4 Department of Health Servicese Research and Policy, Australian National University, Canberra, Australia; 5 School of Health Professions Education, Faculty of Health, Medicine and Life Sciences, Maastricht University, Maastricht, The Netherlands

**Keywords:** GP training, Postgraduate training, Qualitative observational research, Self-regulated learning, Supervisors, Trainees, Workplace learning

## Abstract

Clinical workplaces offer postgraduate trainees a wealth of opportunities to learn from experience. To promote deliberate and meaningful learning self-regulated learning skills are foundational. We explored trainees’ learning activities related to patient encounters to better understand what aspects of self-regulated learning contribute to trainees’ development, and to explore supervisor’s role herein. We conducted a qualitative non-participant observational study in seven general practices. During two days we observed trainee’s patient encounters, daily debriefing sessions and educational meetings between trainee and supervisor and interviewed them separately afterwards. Data collection and analysis were iterative and inspired by a phenomenological approach. To organise data we used networks, time-ordered matrices and codebooks. Self-regulated learning supported trainees to increasingly perform independently. They engaged in self-regulated learning before, during and after encounters. Trainees’ activities depended on the type of medical problem presented and on patient, trainee and supervisor characteristics. Trainees used their sense of confidence to decide if they could manage the encounter alone or if they should consult their supervisor. They deliberately used feedback on their performance and engaged in reflection. Supervisors appeared vital in trainees’ learning by reassuring trainees, discussing experience, knowledge and professional issues, identifying possible unawareness of incompetence, assessing performance and securing patient safety. Self-confidence, reflection and feedback, and support from the supervisor are important aspects of self-regulated learning in practice. The results reflect how self-regulated learning and self-entrustment promote trainees’ increased participation in the workplace. Securing organized moments of interaction with supervisors is beneficial to trainees’ self-regulated learning.

## Introduction

In postgraduate education, trainees mainly practice medicine independently under supervision. Clinical practices offer trainees rich, authentic and varied situations, in which they can learn from experience (Dornan et al. 2007; Stok-Koch et al. 2007; Teunissen et al. 2007b; Watling et al. 2012; Yardley et al. 2012). To learn more consciously self-regulated learning (SRL) skills are important. There is ample literature on SRL, describing definitions, models and stages of SRL, and the cognitive and meta-cognitive processes, such as goal-setting, planning learning activities, self-assessment, and motivation that drive it (Artino and Jones 2013; Boekaerts 1997; Puustinen and Pulkkinen 2001; Sandars and Cleary 2011; Sitzmann and Ely 2011; Zimmerman 2000). A well-known definition comes from Zimmerman, who termed SRL as ‘self-generated thoughts, feelings, and actions that are planned and cyclically adapted to the attainment of personal goals’ (Zimmerman 2000). From a socio-cognitive perspective, an individual reflects on his/her behaviour in the environment and purposefully makes choices on what to do (Artino and Jones 2013; Puustinen and Pulkkinen 2001; Sandars and Cleary 2011; Zimmerman 2000). Self-assessment is important in SRL but error-prone and therefore in need of external calibration (Bjork et al. 2013; Brydges et al. 2010; Regehr and Eva 2006). Therefore supervisors have an essential role in the assessment of trainees. In this respect the literature describes informed self-assessment, facilitated reflection and directed self-guided learning (Brydges and Butler 2012; Brydges et al. 2010; Butler and Brydges 2013; Sargeant et al. 2008; Schumacher et al. 2013). Furthermore, supervisors can support trainees and encourage them to engage in learning activities and reflection (Boendermaker et al. 2000; Brydges et al. 2010; Kilminster et al. 2007; Mann et al. 2001; Sandars and Cleary 2011; Sutkin et al. 2008; Wearne et al. 2012).

In researching SRL in clinical workplaces, it is relevant to consider socio-cultural learning theories as they describe how learning is shaped by participating in practice and by using the affordances that a practice offers (Billett 2004; Bleakley 2006; Durning and Artino 2011; Lave and Wenger 1991; Mann 2011; Pimmer et al. 2013; Swanwick 2005; Taylor and Hamdy 2013). An important concept is legitimate peripheral participation, which is described by Lave and Wenger as a way of learning and developing identity in practice (Lave and Wenger 1991). Newcomers to the practice function at the periphery and move more to the centre of the community over time. They achieve legitimacy as they feel they are members of the workplace and participate in activities that are relevant for the workplace. By performing SRL in clinical workplaces learners might influence their participation in practice and their sense of legitimacy.

SRL often had been studied in formal education contexts. How SRL works in the clinical workplaces, which by their nature refer to informal learning, is rather unclear yet (Brydges and Butler 2012; Butler and Brydges 2013; Schumacher et al. 2013; Sitzmann and Ely 2011). In a previous interview-based study we explored the mechanisms of SRL in a postgraduate training (Sagasser et al. 2012). This study revealed that trainees used a variety of learning activities to tackle medical problems. While trainees learned by repeated activities over a prolonged period of time, they also performed activities just to handle the situation, not to gain a deeper understanding (Sagasser et al. 2012). The literature, however, has demonstrated that this latter type of activities is not necessarily effective for learning, considering the lack of time to answer all aspects or the risk of consulting resources that are within easy reach rather than the most appropriate ones (Coumou and Meijman 2006; Dawes and Sampson 2003; Kortekaas et al. 2015; Morgan et al. 2015). The trainees in this study seemed to choose their learning strategy depending the situation. Woods et al. (2011) also found that the context of a rotation influenced medical students’ choice of learning strategy, rendering differing learning opportunities. In one of our other studies into workplace learning, supervisors reported to be highly committed to trainees’ SRL (Sagasser et al. 2015). However, their attention to trainees’ learning varied, as they discussed all the patients seen by trainees in the beginning, while later on they discussed only those patients that the trainees wished to address. So informal SRL was often undertaken individually and the trainees differed in their timing and approach, inducing the supervisors to offer various degrees of support. The studies showed that both the context and the supervisors influence trainees’ choice of learning strategy, stressing the importance of a socio-cultural learning perspective. However, it is not clear what aspect of SRL contribute to trainees’ learning in clinical workplaces, and how the supervisors contribute to this, regarding the variety of trainee and supervisor activities. We wanted deeper understanding of how trainees regulate their learning from patient problems and how supervisors support this, taking a socio-cultural perspective into account (Li et al. 2010; Lockspeiser et al. 2015; McEwen et al. 2015; Nothnagle et al. 2011; Woods et al. 2011). Such understanding may add to our knowledge of SRL in informal learning situations and may give clues as to how learning in the workplace can be optimised. Our research question, therefore, is ‘What aspects of self-regulated learning contribute to trainees’ competencies as medical expert and what is the supervisor’s role herein?’ We specifically focused on trainees’ SRL activities related to patient encounters. To gain these deeper insights we decided to observe trainees and their supervisors in practice. We conducted a qualitative non-participant observational study in the context of a postgraduate training for general practice (GP).

## Methods

We based our research on the epistemological assumption that multiple truths are constructed by and between people (Bergman et al. 2012; Carter and Little 2007). From this constructive perspective we designed a non-participant observational study. In non-participant observation the researcher is present as an observer but does not participate in the activities being observed (Angrosino 2007; Liu and Maitlis 2010). To focus on the meaning of the experience, we used a phenomenological approach to data analysis (Creswell 1998; Giorgi 2006).

### Context

We performed the study in the first year of the three-year postgraduate GP training in the Netherlands, which is offered at eight University Medical Centres in their departments of general practice/primary care (Van Berkestijn 2002). In years 1 and 3 trainees provide patient care in a general practice under the supervision of a designated supervisor. In year 2 trainees rotate between placements in hospitals, psychiatric outpatient clinics and nursing homes with different supervisors. When trainees experience difficulties in their patient encounters, they can consult their supervisors. Debriefing sessions are organised daily to discuss trainee’s encounters (Boendermaker et al. 2002), whereas educational meetings, which are scheduled one to three times a week, serve to address medical themes and trainee’s development more thoroughly. Reflection and feedback on experience, assessment and personal development planning are methods used in supervision (Pelgrim et al. 2013). All supervisors are experienced general practitioners who attended a compulsory long-term training programme on educational and coaching skills at the university.

### Participants

We invited supervisor-trainee pairs to participate as this allowed us to study both trainees’ activities and the interaction between trainee and supervisor. Inclusion criteria were first-year trainees who had been in practice for at least two months and supervisors who had supervised at least three trainees. We chose to focus on first year trainees as they are novice to general practice and we expect this group to go through an extensive learning process. First, we invited supervisors in writing and by telephone. Upon their acceptance, we approached their trainees. When they agreed, we made appointments for observations. Before the collection of data, supervisors and trainees gave written informed consent.

### Design, data collection and triangulation

We obtained data from observations, interviews and documents, thereby promoting data triangulation (Mays and Pope 2000; Tavakol and Sandars 2014). One researcher (MS) visited each pair on two separate days with an interval of one to three days. The participants were informed that the study aimed to get insight into how trainees learn from patient encounters, debriefing sessions and educational meetings. The practice had informed patients verbally and in writing about the researcher’s presence and all observed patients gave prior written consent; when they had not, MS would leave the consultation room. MS encouraged the trainees and supervisors to act as they normally would. To capture the origin of trainees’ learning process, MS observed trainees manage patient encounters. Her observations focused on trainees’ activities before, during and after the encounter that could indicate that the trainees did not know enough to manage the encounter. MS took field notes on health problems presented, patient characteristics, trainees’ activities, materials used, trainees’ interaction with others (e.g., supervisor), and the consultation room, for example. To keep track of the trainees’ learning process, MS observed the patient debriefing sessions and educational meetings with supervisors, which were audio-taped, transcribed verbatim and de-identified. Supervisors and trainees determined when these patient debriefing sessions and educational meetings took place. Field notes on the encounters, patient debriefing sessions and educational meetings were typed out and de-identified shortly after observation. On the second day, after the observations, MS interviewed trainees and supervisors separately. She asked trainees to reflect on their self-regulated learning from the observed encounters and on the contribution of the patient debriefing sessions and educational meetings herein. Other interview topics concerned trainees’ prior experience as a doctor, the workplace and the university. Supervisors were asked about their role in trainees’ learning, especially during the encounters, patient debriefing sessions and educational meetings. All interviews were audio-taped, transcribed verbatim and de-identified. Supervisors and trainees received a gift coupon for their participation. Our research team included one educationalist (MS) and four experienced researchers and educators with differing backgrounds: two general practitioners (AK, CvW), a psychologist (CvdV) and an educationalist/medical doctor (CF).

### Ethical approval

The Ethical Review Board of the Netherlands Association for Medical Education (NVMO; no 368) approved the study. Anonymity was guaranteed as transcripts and field notes were de-identified. Participation was voluntary.

## Data analysis

**Fig. 1:**
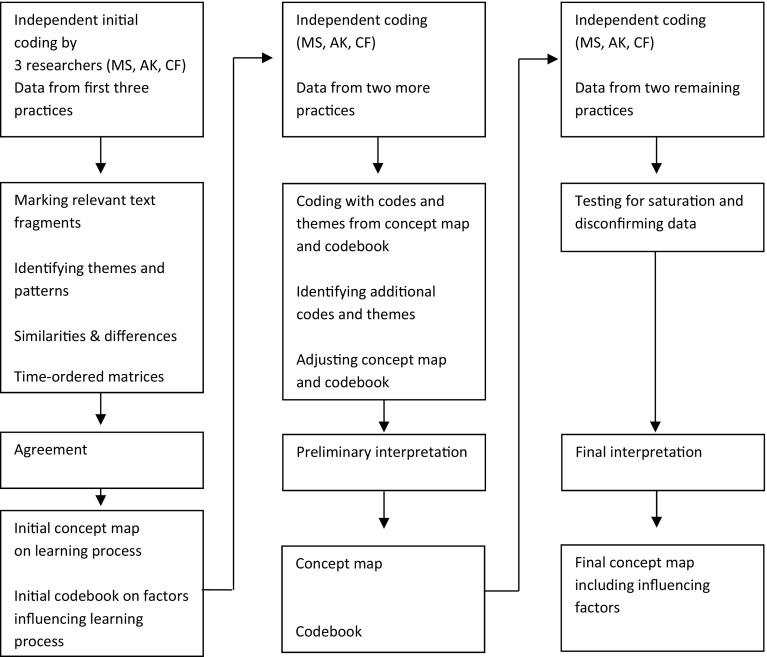
Analysis process

Three researchers (MS, AK, CF) performed a qualitative analysis of the data, using a phenomenological analytic method (Angrosino 2007; Creswell 1998; Giorgi 2006). The method involved a search for themes and patterns and allowed for interpretations. The analysis consisted of independently reading and rereading, marking relevant text fragments, identifying and coding themes and patterns, and discussing these findings in the research team, which resulted in a description of findings. During the analysis, the researchers critically reflected on their differing backgrounds which brought various perspectives to the data, thereby promoting reflexivity and confirmability (Barry et al. 1999; Mays and Pope 2000; Tavakol and Sandars 2014). We iteratively collected and analysed the data, starting the analysis as soon as the first data became available, which technique bolstered dependability (Mays and Pope 2000; Tavakol and Sandars 2014). During the analysis MS kept a reflective diary, the review of which enhanced our understanding of observations. The analysis was an inductive process consisting of three consecutive phases (Fig. 1). First, we independently read and reread the data pertinent to the first three practices. During analytic sessions we discussed and searched for relevant themes. We developed time-ordered matrices describing per encounter trainees’ activities before, during and after the encounter, supervisors’ related activities, the related dialogue in the debriefing session, and supervisors’ and trainees’ accounts in their interviews (Miles et al. 2013). We searched for similarities and differences between the processes and for possible explanations. We also explored how educational meetings related to the encounters. We drew an initial concept map to bring this learning into focus (Miles et al. 2013). We developed an initial codebook for factors influencing this learning (Miles et al. 2013). Second, we analysed data from two more practices, and we verified whether the data could be organised by the concept map and the codebook. Continued interpretation resulted in refinement of the concept map and codebook. Finally, we analysed the data of the two remaining practices to ensure saturation. We carried out member checks by sending the preliminary results to trainees and supervisors, thereby enhancing credibility (Mays and Pope 2000; Tavakol and Sandars 2014).

## Results

**Fig. 2:**
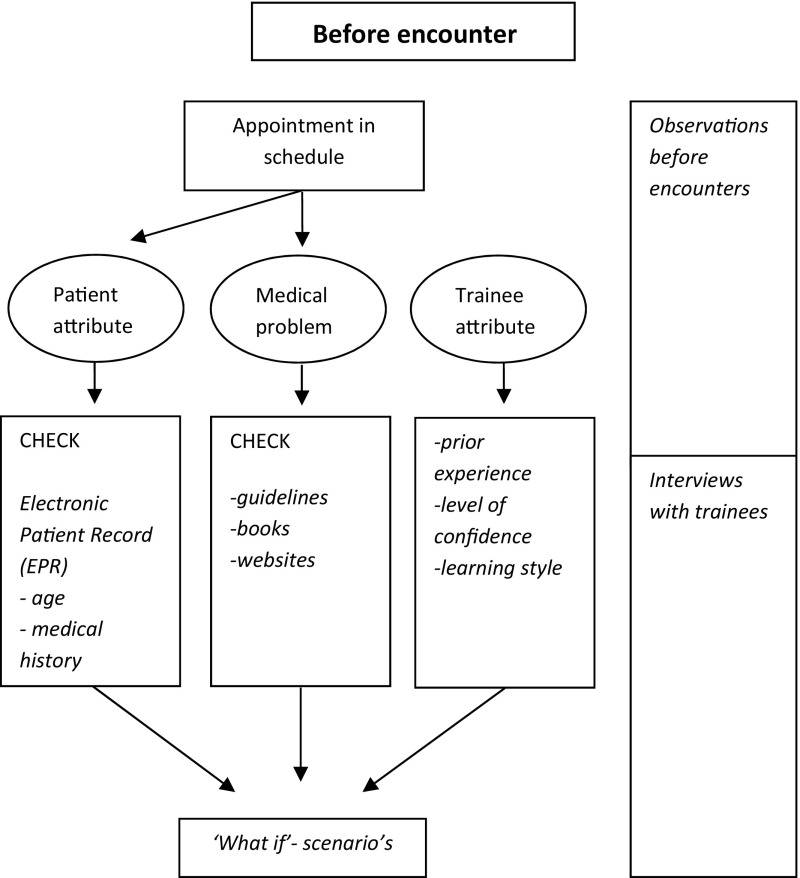
Activities before the encounter. The *right-hand* column indicates from which sources the data were derived

**Fig. 3:**
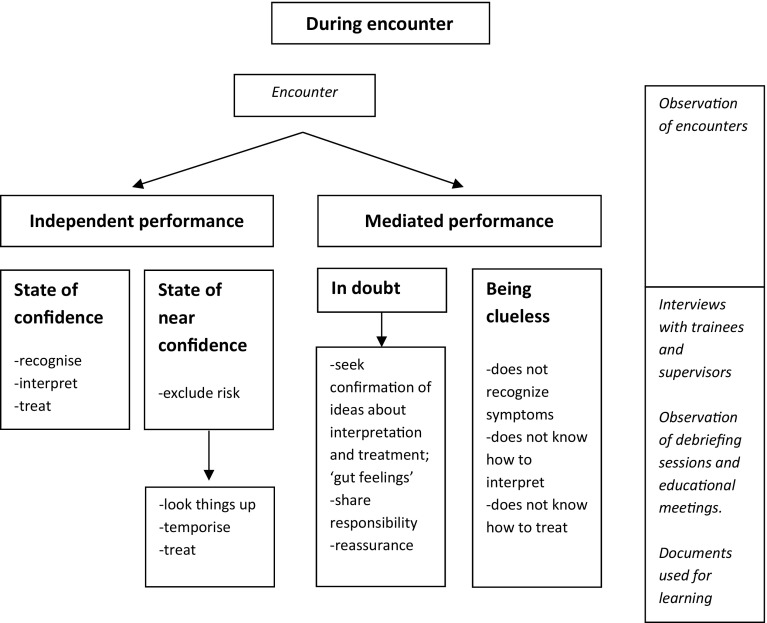
Activities during encounters that contribute to trainees’ learning. The *right-hand column* indicates from which sources the data were derived

**Fig. 4:**
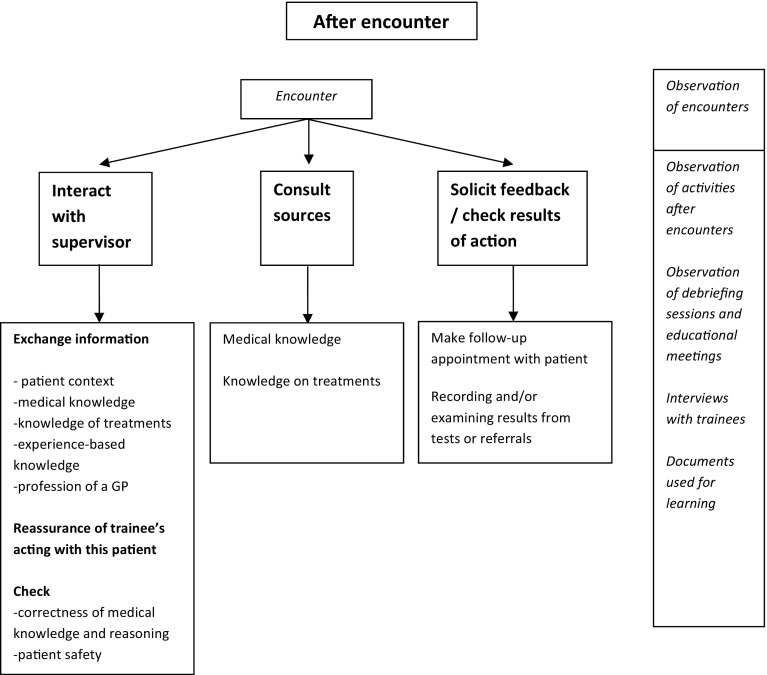
Activities after encounters that contribute to trainees’ learning. The *right-hand column* indicates from which sources the data were derived

We collected data from November 2014 to March 2015. Seven supervisor-trainee pairs participated. Six supervisors were male and all trainees were female. Supervisors’ mean age was 53 years (range of 44–64) and trainees’ mean age was 29 years (range of 26–40). MS did not know the participants before. From the seven practices we included 112 patient encounters in total, varying from 5 to 40 min in length. Some encounters featured multiple symptoms or multiple patients. Five encounters could not be attended, since patients did not want the researcher to be present. Debriefing sessions lasted 26 min on average, educational meetings 41 min. Interviews with trainees took 45 min on average, those with supervisors 38 min. We will present the results by describing trainees’ activities related to patient encounters and trainees’ and supervisors’ interactions. Figures 2, 3 and 4 map out trainees’ activities before, during and after encounters respectively, and the data that informed these results. We observed that trainees managed most of the encounters independently, that they occasionally consulted their supervisors during the encounters, and that most of the encounters were discussed during debriefing sessions.

### Before the encounters

We observed that, before the encounters, trainees used the electronic patient record to inform themselves about patients and their medical problems.

“Sometimes there are follow-ups, so you want to see what these patients came for earlier, sometimes you saw patients yourself, sometimes other doctors saw them, then you want to know what has been done or what has been planned for a next visit, as patients will have certain expectations. Sometimes patients show up with new complaints, and I want to know the history, what medication they use, so I can respond properly to them.”(Practice W, trainee interview).

Incidentally, trainees consulted family practice guidelines or other sources of medical knowledge. In the interviews, trainees explained that they used these sources not only to gain more knowledge about medical problems, diagnostic steps or treatments, but also to ascertain whether their first ideas about these medical problems and diagnostic steps or treatments to be taken were correct. In order to be prepared for the patient’s possible problem presentation or patient’s questions, some trainees used such information to develop ‘what-if’ scenario’s.

“Basically I use the medical guidelines, to know what steps to take, because you know the patient’s complaints. (..) That I know what examination I could do and.. what íf..that I have an action plan in my head, it may be quite short because I can also look things up during the encounter.” (Practice D, trainee interview).

### During the encounters

#### Managing the encounter without supervision

We observed that trainees managed most of the encounters independently, but also regularly looked things up during the encounters. From the interviews it became clear that this independence was accompanied by either a sense of confidence or a sense of near confidence, indicating that trainees were engaged in a process of self-entrustment. When feeling confident, trainees explained that they recognised the medical problem, knew how to interpret the symptoms and felt they could act independently. Often, however, trainees reported a feeling of near confidence, meaning that they were not entirely sure how to interpret or treat the symptoms or they felt they did not know enough about the medical problem. Yet, they felt they could initiate treatment without their supervisor’s mediation if they could exclude serious medical problems and were able to devise a therapeutic plan, with the aid of information sources or otherwise.

“Mmm, the patiënt with that eczemalike symptom, yes, I did not exactly know what it was. Um, when it comes to skin diseases I really have to make an effort, yes, I find it difficult, a lot of things look alike, or just do not look quite typical. In this case I consciously chose not to consult the supervisor, because sometimes I have to, yes, I have to act. By trial and error that is. As long as it doesn’t harm the patient.” (Practice R, trainee interview).

“Um, oh yes, with that second patient, that child, umm, I was not really in doubt, but I did find her ill, but she was very vivid, so I was in doubt to let her [=supervisor] observe, but still, no, no, this decision I could make for myself (..) I thought, yes, I felt I could decide that it was all right.” (Practice P, trainee interview).

Trainees explained that they sometimes, provided the patient would not be put at risk, purposefully temporised treatment by planning a follow-up encounter and meanwhile informing themselves about further treatment.

#### Managing the encounter with supervision

We observed that trainees occasionally consulted their supervisors during the encounters. From the interviews we learned that trainees consulted their supervisors when they were ‘in doubt’:

“Interviewer: What was your reason for calling the supervisor?

Trainee: Well, the patient had no history of asthma. If he had asthma or COPD and low CRP levels, I would have given him prednisone, but since he had no history of any of these conditions I thought… well … should I give him an inhaler or prednisone? Prednisone is better to treat chronic infections (..) and an inhaler when the airways are um..(..). But the story was not clear, he had none of these symptoms.” (Practice F, trainee interview).

Sometimes, trainees consulted their supervisor to verify whether the diagnosis or treatment they had in mind was correct. This was especially the case when trainees identified the problem as risky or had a premonition of something being wrong. Example 1 in Table 1 illustrates a trainee’s consideration about an encounter and a supervisor’s role herein with data from field notes, and trainee and supervisor interviews.

**Table 1:** Examples of how various data inform on trainee’s and supervisor’s roles and activities in trainee’s learning from patient encounters

Example	Type of data	Data
Example 1 Practice P	Field note encounter	The trainee sees a 91 year old woman, who is suffering from a severe cold for two weeks. She uses a nose spray. She has got red-brown snot drips out of her nose, and watery eyes. The trainee asks questions regarding the cold. The patients says to have a sore throat as a result of coughing. The coughing is painful in her whole body. After having taken the history the trainee examines the patient. She examines heart, lung, nose and ears, and measures the oxygen saturation. Then the trainee (T) says to the patient (P): “T: I ask doctor S. to have look at your neck, it’s a bit swollen. P: During a surgery for breast resection I had a tube, they touched something there.” The trainee calls her supervisor on the phone, and tells what she found *‘right behind the clavicula a thickened gland, but left as well’*. She asks her supervisor to examine as well. The supervisor comes in and examines the patient. The supervisor(S) starts a dialogue with the trainee. “S: it looks bigger. I don’t think it’s the gland. I would…what would you do? T: X-ray, or ultrasound. S: What’s the easiest? T: X-ray. S: Why wouldn’t you do both? T: You want to know the problem, you can see it with ultrasound; X-ray can provide additional information.” The supervisor leaves the consultation room.
	Trainee interview	“Trainee: It was that gland, I actually already knew it was not okay (..) it had nothing to do with the reason for her visit, it was an unexpected finding. I already saw it when she was sitting in front of me, one big gland is bad news. Considering her history she also had a thickening on the other side, so that’s why I began to hesitate. You see, if she had one big gland on this side, you would know immediately, but then I started hesitating. Interviewer: That’s why you asked your supervisor to come in? Trainee: Yes, to review my physical examination, because she had thickenings on both sides.”
	Supervisor interview	“I have to be aware not to take over. When she consults me during the encounter I have to ask her what she would do. The encounter with the older lady with that gland, I did not say ‘Go make an X-ray and an ultrasound’, but I asked her ‘What would you do?’ (..) I asked her ‘What’s the best choice’ (..) On that moment I want her to consider what test would be the most informative and should be requested. She will learn the most when she’s involved in it. I discuss it briefly with her in presence of the patient, as we are actively involved. (..) So I have to be aware, it’s her moment to learn, so she should come up with an answer.”
Example 2 Practice N	Field note encounter	The trainee sees a woman with climacteric complaints. The woman says that earlier the general practitioner [who is trainee’s supervisor] prescribed pills but that her complaints came back. She has trouble sleeping because of hot flushes and restless legs. She’s very tired. The trainee says that she wonders whether the sleeping problems and restless legs indeed are related to the menopause. She says that she wants to do a hormone determination to check this. The patients asks for sleeping medication and says that she had sleeping medication before. The trainee explains that sleeping medication is just a remedy, not the solution. The trainee prescribes sleeping medication for 10 days, and writes a referral for a blood test, and asks the woman to make a follow-up appointment for the results of the blood test.
	Debriefing session	Trainee and supervisor read in the electronic patient record what the trainee wrote about the patient. “Trainee: Well, first she started with hot flushes, climacteric complaints, but eventually she asked for sleeping medication. But I thought it’s not only the hot flushes, there’s much more (..) because of the way that she told it. I had no clues. During this encounter I could not extensively question her, I think a second encounter is necessary.(..) I thought I do a blood test to exclude physical causes, and then I can question her more.. Later I saw in her record that she has some difficulties at home. (..) Supervisor: Well, I think that you sensed very well that there’s much more at stake than just uhm the request for pills. Because, that’s indeed not the solution for the problem, there’s much more behind it. When I hear this story, indeed, I think ‘What does she really want?’ Does she just want the sleeping pills because she can’t sleep and is it all right then, or does she want to visit the psychologist again? (..) Yes, it’s a tough, it’s a difficult, complex problem.”
	Trainee interview	“Trainee: This patient will come again probably, it’s only the beginning of a process. Especially such climacteric complaints are chronic, they are not solved within a week. I wanted to discuss her with S. (Supervisor), because in her record I saw that she also had sleeping problems and stress. (..) So I took these into consideration ‘Are her complaints only climacteric or is something else at hand? Because I do not know this patient I have to instantly evaluate her. How do I do that? You see, if she only wants a solution for hot flushes, I would prescribe something, and then I would finish the encounter, and the patient would go home with medication. But I was wondering if that was her question. Interviewer: How did the debriefing session help you? Trainee: I got reassurance, that I was on the right track, that I saw it right and made a good evaluation. S. knows this patient, so you ask for reassurance ‘Is it right what I saw or noticed on this woman?’.”

Trainees also explained that they consulted their supervisors when they were clueless, that is, when they did not recognise or know how to interpret the symptoms. This lack of knowledge, however, did not show during the observed encounters. Finally, trainees consulted their supervisors to share responsibility, which they especially valued when decisions, for instance about serious problems, referrals, X-rays or costs, had important consequences for the patient. Sometimes trainees also consulted supervisors to reassure patients.

### After encounters

Our observations of the debriefing sessions and educational meetings, as well as the interviews with trainees and supervisors revealed that interaction with supervisors helped trainees to expand their expertise on illnesses and treatments, to discuss their professional development and to guarantee patient safety. The debriefing sessions, which followed shortly after the encounters, fulfilled three purposes. First, they served to respond to informative questions from both trainees and supervisors. The trainee and supervisor exchanged and discussed contextual information about patients, different manifestations of medical problems, medical and evidence- and experienced-based knowledge, and possible treatments.

“Trainee: (..). But it shows how much it affected him. Would you have given him something else, would you have given him tramadol?

Supervisor: No, no

Trainee: Yes, I think that would make him sleepy

Supervisor: No, I do not favour tramadol

Trainee: No, I do not like it either

Supervisor: I think it is poison. Don’t like it at all. Half of the people can tolerate it, and for the other half it is a burden because they cannot tolerate it

Trainee: No, tramadol is not that effective, as research shows.”

(Practice R, debriefing session).

“Supervisor: You usually begin high and then you reduce it. With 1 mg you may or you may not get there. So I usually give 3 or 5 melatonine (..) and then see for two weeks if it works. If after two weeks 3 mg does not help, you don’t have a melatonine insufficiency and you’d better stop. And with 1 mg you just can’t be sure, because it’s right on the edge.” (Practice K, debriefing session).

Second, the debriefing sessions served to reassure trainees. Trainees’ sometimes asked for such reassurance when reporting on encounters they performed independently, or when reflecting on encounters during which they consulted their supervisor. Example 2 in Table 1 illustrates such reassurance with data from field notes and interviews. Sometimes supervisors expressed reassurance spontaneously while trainees had not asked for it.

“Supervisor: What you do is okay, asking for a follow-up. One week of amplodipine is too short to examine that.”(Practice F, debriefing session).

During the interviews both supervisors and trainees clearly mentioned the importance of reassurance in the debriefing sessions.

“Erm, resolving uncertainty a bit (..) that I can share and that he [supervisor] says ‘ok, this is fine’, or ‘no, you better do something else’, yes, resolving uncertainty.” (Practice W, trainee interview).

“Well, when it’s clear-cut I feel I can decide myself. When it’s not as expected I incline to ask my supervisor what he would do. But if there isn’t anybody around I can decide myself, but it’s just that reassurance, I mean, he [supervisor] hardly says something completely different from what I suggest. That’s why my supervisor says ‘I don’t understand why you want to discuss all those patients with me, because you never do anything wrong.’ But I may be insecure, that’s why I ask for his reassurance.” (Practice F, trainee interview).

“I say to her, do you at this moment have patients that cause bellyache or sleepless nights or whatever, we have to discuss them, we just have to. (..) In the beginning, I notice, there’s great uncertainty.” (Practice K, supervisor interview).

Finally, for both supervisors and trainees the debriefing sessions served to check on trainees’ performance, knowledge and skills.

“Ermm, well, to check a little, for myself. Uh, to hear what she is able to do and how she performs. Especially her thinking process, (..), to hear how she does the encounter and eventually comes up with a solution.” (Practice R, supervisor interview).

By reporting on patients trainees gave their supervisors insight into their performance and showed them to take good care of the patients.

“I report things just to have mentioned my decisions. (..) It’s just open reporting of what you did and when there are no comments, it was all right. To me it’s some sort of check whether I did the right thing, it’s the unmentioned question ‘Do you agree with my decisions?’.” (Practice K, trainee interview).

When trainees reported on patients, supervisors would ask questions to ensure patient safety and gauge trainees’ medical knowledge, reasoning and decision-making skills.

“What people are most likely to develop complications? ‘and ‘How is otitis externa treated?’” (Practice N, debriefing session).

We also observed educational meetings during which trainees and supervisors discussed medical themes (such as eczema, depression, asthma/COPD) more thoroughly, aimed to deepen understanding and to apply this knowledge to a broader population. Other topics under discussion were guidelines, consultation skills in video-taped encounters and trainees’ development.

We observed that trainees consulted various sources after the encounters, such as books, guidelines or web-based information. Trainees explained that they looked for information about treatments or medical knowledge, or for a confirmation that their decisions were, indeed, correct.

“When someone [a patient] asks me a question, I will answer it but then I just know when I’m not entirely sure. Those are the things I tend to look up, I find, I have to look them up to know for sure. (..) And after I’ve seen patients, I like to consider and to check, with those specific patients in mind: was I right to do something in this patient but not to do it in that one? You hope to develop some sort of range of patients to whom such treatment is applicable.” (Practice K, trainee interview).

Moreover, trainees reported to be active in soliciting feedback on their performance, which they found meaningful. For instance, they learned from follow-up encounters in which they could witness the effects of their treatment and the development of the medical problem. Similarly, reviewing incoming emails concerning referrals or laboratory test results appeared to be a powerful learning activity. Trainees felt that their medical expertise grew by connecting these results to their expectations of the medical problem.

“In practice the trainee reviews incoming email about patients (referrals or tests). She explains that she wishes to know the results from the patients she treated in order to find out whether her expectations were correct. She says ‘Oh, see, I referred this patient, I like to read it, it was an ankle problem.’ She says that when the results are different from what she expected, she will discuss it with her supervisor.” (Practice R, field notes).

Sometimes, to maximise their clinical experience, trainees also applied this procedure to patients they had not seen themselves.

The workplace also offered trainees other opportunities for spontaneous information exchange and discussion. We observed, for example, that during coffee breaks supervisors, trainees and other health workers exchanged medical or contextual information of home visits.

## Discussion

Trainees’ self-regulated learning from patient encounters is a dynamic process. Trainees evaluate whether they know enough to manage the encounter and whether they feel confident enough to manage the encounter independently. They actively seek information, confirmation and feedback, engage in reflection and consciously decide whether to consult their supervisors. Supervisors have a key role in this process, as they foster learning by exchanging and discussing experiences, providing knowledge, reassuring, providing feedback and checking trainees’ knowledge and skills. Our results illustrate how trainees’ SRL contributes to their self-perceived growth of competence. In the following paragraph we will elucidate the role of confidence, reflection and feedback, and the supervisor, and discuss our results in view of socio-cultural learning theories and legitimate peripheral participation.

Since the workplace not always offers a patient mix attuned to their level of competence (De Jong et al. 2011), trainees have to handle all kinds of patients problems. Trainees in our study reflected whether they felt confident to manage the patient problem. This confidence relates to the concept of self-efficacy that is often described as important in SRL. Self-efficacy refers to trainees’ beliefs in their ability to perform training-related tasks (Artino 2012; Bandura 1986; Dory et al. 2009). Successful performance in similar previous situations promotes self-efficacy. Self-efficacy often refers to specific tasks. Patient encounters, however, often contain multiple specific or nonspecific and possibly risky tasks that have to be performed simultaneously. Therefore, we coin the term self-entrustment to refer to the process in which trainees evaluate to perform independently taking patient safety into account. Trainees experience their increase in self-entrustment as an indicator of their self-perceived growth in competence.

During the encounter trainees in our study reflected on what would be necessary to proceed. Such reflection ‘in the moment’ has been termed by Schön as ‘reflection-in-action’ (Schön 1983). Trainees actively used various ways to inform themselves. For instance, they searched for feedback on their performance by assessing results of their patient care and by discussing their performance with their supervisors. They related such feedback to their own knowledge, skills and competencies and, in interaction with their supervisor, to broader evidence-based or experience-based knowledge. In interpreting such feedback, trainees engaged in individual reflection and, with their supervisor, in interactive reflection. The importance of reflection and feedback in our results is consistent with the literature on SRL and with the literature on learning from clinical work (Sandars 2009; Teunissen and Bok 2013; Veloski et al. 2006; Watling et al. 2012). Repeatedly seeing patients, receiving feedback and reflecting on performance contributed to trainee’s self-entrustment and to their self-perceived growth in competence.

The finding that supervisors play a vital role in trainees’ self-regulated learning is consistent with the literature on supervision (Brydges et al. 2010; Kilminster et al. 2007; Sutkin et al. 2008; Wearne et al. 2012). Supervisors confirmed trainees’ performance, helped them to reduce uncertainty, develop knowledge and skills in the GP context, and were alert to the inherent risk of the trainee being unconsciously incompetent which could harm patients (Van Mook et al. 2015). Supervisors needed to have confidence that their trainees would consult them when needed, while trainees, in turn, needed to be able to count on their supervisors for support (Hauer et al. 2014; Kennedy et al. 2007). To enable such supervision a good working relationship between supervisor and trainee was a prerequisite. Sufficient contact time over time, like debriefing sessions, educational meetings and other moments in the workplace, is necessary to develop and maintain such a relationship (Hirsh et al. 2007; Watling et al. 2010).

From a socio-cultural learning perspective our results illustrate how trainees, in using their SRL skills, increasingly participated independently in patient care in the GP practice, and thereby reflect legitimate peripheral participation (Lave and Wenger 1991). The trainees in our study actively participated in the practice, and used their agency for making choices to act independently or not. Agency refers to a person’s power to act, to become an independent actor (Bandura 2001). Repeatedly exercising SRL activities bolstered trainees’ self-entrustment and their self-perceived growth in competence, and it increased trainees’ legitimacy and promoted their move to the centre. Trainees in our study expressed their legitimacy as being able to act independently and being part of the practice, while also feeling assured not to have all competencies yet.

Our findings also relate to studies that investigated learning in clinical workplaces from a socio-cultural perspective. Van der Zwet et al. (2011) found the importance of developmental space in order to learn and to develop a professional identity for students in a GP clerkship. Within this space students developed their legitimacy and moved to the centre of the community. The importance of inclusion in workplace social practices was also found by Strand et al. (2014) as they identified ‘learning as membership’ as one of supervisors’ conception of trainee’s learning. Strand et al. also found the importance of interaction with the supervisor by identifying ‘learning as partnership’, which refers to a process of collaborative meaning making between supervisor and trainee. Teunissen et al. and Clement et al. also found the importance of such interaction (Teunissen et al. 2007a; Clement et al. 2016). As in our study, the concept of agency is reflected in the studies from Van der Zwet et al., Strand et al. and Teunissen et al., as the trainees and students take an active role in their learning (Van der Zwet et al. 2011; Strand et al. 2014; Teunissen et al. 2007b).

We found that trainees varied in what patients they looked up information for, consulted their supervisors for or discussed about with their supervisors. This may be explained by trainees’ differences in level of competence and level of confidence. These influenced what trainees perceived as difficult and influenced their choice of action. While some trainees found specific patient problems difficult, like breathing problems, others did not because of their prior experience. In terms of legitimate peripheral participation, trainees were more central in patient care for some patient problems than for others.

In reflecting on our results, we found how using their SRL helped trainees to become legitimate participants in practice and we found how the practice afforded trainees to use their SRL. Various aspect can be organized and supported in practice. Trainees need space to perform independently and to take responsibility for their performance, and they need supervisor’s trust in this respect. It should be clear to trainees how and when they can consult their supervisors. Having the supervisor nearby facilitates instant consultation and promotes their learning. Scheduled appointments or routines in the practice for debriefing sessions or educational meetings secure moments for interactive reflection and meaning making. Access to sources of information, like computer facilities, books or other persons, is essential in trainees’ learning.

Our study had some strengths. The observational character provided rich and varied data allowing us to take holistic views on learning in the workplace. The variety of data promoted triangulation. The data derived from observing trainees and supervisors, both individually and in interaction with one another, provided insight into actual behaviour. The interviews gave insight into participants’ thoughts about their behavior. Moreover, this study adds to evidence we found in previous research and provided a deeper understanding of trainees’ self-regulated learning from patient encounters (Sagasser et al. 2012, 2015). However, there were also weaknesses. The most important weaknesses of this study were the researcher’s presence and the fact that the participants were informed on the aim of the study. These may have influenced their behaviour. Despite our encouragement to act as they normally would, trainees and supervisors may have behaved differently. In interviews there may also have been a discrepancy between what people said and what they actually did. The combination with observational data reduces this weakness. Supervisors and trainees participated voluntary and therefore may have been more interested in self-regulated learning or workplace learning than others. Furthermore, we failed to include more female supervisors and male trainees. However, this male supervisor to female trainee distribution largely reflects reality. This study focused exclusively on learning from patient encounters, eliciting mainly competencies in relation to the roles of medical expert, communicator and professional. Self-regulated learning in relation to other competencies may evolve in another way. We studied first year trainees and their supervisor in general practice. The results depended on the autonomy that supervisors granted the trainees. Our results may not be easily transferable to more advanced trainees and other specialty training contexts where autonomy for trainees and the trainee-supervisor relationships are different.

In conclusion, our study adds on the literature on SRL related to workplace learning by giving insight into how SRL promotes legitimate peripheral participation in the workplace. Our study demonstrates how trainees actively seek information, confirmation and feedback, how they use opportunities to learn in the workplace, how they engage in individual and interactive reflection, and how self-confidence affects trainees’ decisions to provide patient care independently. We introduce the term self-entrustment in this respect. Supervisors are vital to this learning process and a good long-term working relation is indispensible. Future research might elaborate on the concept of self-entrustment as a central mechanism in self-regulated learning in the workplace. Furthermore, deeper insight into the interaction between supervisor and trainee may enhance our understanding of workplace learning.
